# Self-Powered Triboelectric Insole for Gait Asymmetry and Plantar Pressure Signatures in Rehabilitation Patients: A Cross-Sectional Study

**DOI:** 10.3390/s26103191

**Published:** 2026-05-18

**Authors:** Perizat Kanabekova, Adeliya Anash, Pedro Morouco, Bekzhan Pirmakhanov, Gulnur Kalimuldina

**Affiliations:** 1 Department of Mechanical and Aerospace Engineering, School of Engineering and Digital Sciences, Nazarbayev University, Astana 010000, Kazakhstan; perizat.kanabekova@nu.edu.kz (P.K.); adeliya.anash@nu.edu.kz (A.A.); 2 Mirai Technovation Ltd., Astana 010000, Kazakhstan; pirmakhanov.b@gmail.com; 3 National Program for Physical Activity Promotion, Directorate-General of Health, 1049-005 Lisbon, Portugal; pedromorouco@dgs.min-saude.pt; 4 Department of Epidemiology, Biostatistics and Evidence-Based Medicine, Faculty of Medicine and Health Care, Al-Farabi Kazakh National University, Almaty 020000, Kazakhstan

**Keywords:** biomechanical analysis, gait analysis, self-powered sensors, triboelectric nanogenerator, lower limb

## Abstract

**Highlights:**

**What are the main findings?**

- Self-powered triboelectric insoles enable battery-free gait monitoring and analysis.
- TENG insoles with IMU sensors monitor spatiotemporal gait parameters, which allow for an analysis of the gait to provide information such as cadence, gait asymmetry, plantar loading and flatfoot.
- Sensor-derived plantar-loading signatures reveal condition-specific gait phenotypes.
- Age and fracture history show a measurable association with waling cadence.

**What are the implications of the main findings?**

- These insoles enable continuous, power-independent monitoring for early detection of gait abnormalities in clinical trials, rehabilitation, and daily use for patients with flatfoot or post-fracture issues.
- The insoles support personalized interventions like custom orthotics by quantifying age- and history-related cadence declines, improving outcomes in pharmaceutical and medical device development.

**Abstract:**

(1) Background: Gait analysis technologies have advanced; however, traditional systems like optical motion capture are lab-bound and costly, limiting rehabilitation monitoring. This cross-sectional study evaluates self-powered triboelectric nanogenerator (TENG) insoles combined with IMU sensors to assess gait asymmetry, plantar pressure signatures, age effects and injury history in rehabilitation patients, aiming to enable portable, battery-free phenotyping. (2) Methods: Fifty-three patients (22 females, 31 males; age, 29 ± 26 years) from Astana clinics with trauma histories (e.g., spine, ankle, fractures) and 10 healthy references underwent a 2 min walk test (2MWT). TENG insoles captured plantar loading; ankle/knee IMUs measured spatiotemporal parameters (cadence, asymmetry). The data were normalized; the analyses used an ANOVA and correlations (Python 3.14.3). (3) Results: The TENG sensors showed force/frequency linearity (up to 10 V at 20 N). The cadence averaged 101 ± 10 steps/min, declining with age (r = −0.31, *p* = 0.03) and fractures (r = −0.23, *p* = 0.04). The asymmetry varied (−54% to +31%) without category differences. Flatfoot (55%) was linked to lateral loading shifts; condition-specific waveform signatures emerged (e.g., lateral heel in ankle issues). (4) TENG-IMU systems feasibly capture gait phenotypes in heterogeneous cohorts, supporting out-of-lab monitoring for personalized rehabilitation without batteries. Prospective validation is required for further practical implications.

## 1. Introduction

Technologies for gait analysis have advanced significantly over the past decade [1]. Currently, various systems (e.g., motion capture, instrumented walkways, wearable sensing systems) aid the detailed evaluation and characterization of gait patterns and measurable parameters. Presenting information on joint kinematics and spatial and temporal gait parameters provides essential biomechanical insights into the joint, which can be used to predict falls [2] or sports injuries [3]. It can also identify the cause of injury, which provides insights into treatment, can be used in rehabilitation and sports, and serves as a diagnostic tool for various diseases [4]. Software facilitates measurement, while various artificial intelligence models and machine learning systems enable precise data interpretation [5].

Abnormalities in gait analysis can also correlate with age-related changes [6]. Using gait analysis data from older adults, it is possible to evaluate the fall and injury risk. Traditional commercial devices such as optical motion capture systems (3D measurement systems, e.g., ViconTM) are considered the “gold standard” due to their high accuracy. However, they are limited by the restricted areas captured, significant dependence on laboratory environments, and high costs of the settings [7]. Therefore, novel approaches are being studied and developed to avoid such restrictions, particularly for on-the-go, portable monitoring of health deterioration and/or progress.

Although sensing technologies are not new to biomedicine, several key sensor classes are already widely used and provide complementary information on mechanical loading and movement patterns. Among these, pressure sensors were among the earliest and most widely adopted, enabling the quantification of applied forces and deformation in clinical and wearable contexts [8]. Overall, flexible, self-powered sensors have recently attracted increasing attention for healthcare applications [9]. In recent years, piezoelectric nanogenerators (PENGs, which convert mechanical energy into electrical signals) have evolved from primarily energy-harvesting devices to platforms that can also operate as medical sensors [10]. In parallel, triboelectric nanogenerator (TENG)-based sensors exploit contact electrification and electrostatic induction to both harvest energy and detect motion-related signals [7]. Collectively, these self-powered approaches support personalized healthcare monitoring by enabling continuous, real-time data acquisition that can inform clinical decision-making and improve patient outcomes.

One of the studies reported a wearable TENG-based system for monitoring gait and waist motion, achieving a nearly 99% identification accuracy and enabling IoT-connected, immersion-enhanced rehabilitation via real-time robotic and virtual-game control, highlighting TENG’s potential in smart, interactive healthcare [11]. Moreover, there is a system based on a hybridized electromagnetic–triboelectric nanogenerator (HETNG) that generates biomechanical energy during human balance control and walking, enabling both motion monitoring (e.g., squatting, standing, leg lifting, and fall detection for the elderly) and powering portable electronics [12]. The potential of TENG in a rehabilitation monitoring system with machine learning was described by Liu et al., 2024, who reported a high accuracy in motion recognition and rehabilitation assessment [13]. Similarly, Demircan et al. (2025) evaluated a TENG-based system that can recognize five distinct activities (walking flat, walking upstairs, walking downstairs, running, and jumping) with a 93% classification accuracy [14]. Another study described a machine-learning-driven TENG insole system with nylon nanofibers that achieved highly accurate user identification and real-time gait monitoring for the detection of pes planus [15].

In this study, we analyzed gait biomechanics across patients in different age groups and with different histories of trauma-related medical conditions using self-powered TENG insoles and ankle- and knee-mounted inertial measurement units (IMUs), which are accelerometers with gyroscopes or magnetometers providing data such as the angular velocity or acceleration. Our aim was to quantify spatiotemporal asymmetry and foot-loading patterns during walking to identify clinically relevant gait phenotypes. More specifically, we sought to (i) characterize age-related differences in cadence and temporal gait symmetry, (ii) examine whether a history of an ankle/foot injury is associated with increased asymmetry compared with other musculoskeletal histories, and (iii) explore how the sensor-derived foot loading distribution (including a center-of-pressure proxy) differs in participants with features consistent with reduced medial arch support. These criteria provide an objective basis for stratifying heterogeneous rehabilitation populations and for prioritizing individuals for a more detailed biomechanical assessment or targeted rehabilitation interventions in future prospective studies.

## 2. Materials and Methods

### 2.1. Study Design and Participants

This was a cross-sectional study of patients with different trauma-related medical histories ranging from major spine injuries to minor ankle injuries, with different times after injury ranging from 3 weeks to more than 5 years. Fifty-three participants (22 females, 31 males; age range, 9–68 years) were recruited from medical clinics in Astana, Kazakhstan. The inclusion criterion was the ability to walk independently for at least 5 min without assistive devices; the exclusion criteria included acute pain at the time of assessment, neurological disorders, or a cognitive impairment affecting gait. Demographic characteristics of the participants described in Table 1. 

In addition, a convenience sample of healthy adults (n = 10; 4 F: 6 M; age, 20–30 years) with no current musculoskeletal pain and no self-reported history of a lower-limb injury or surgery was recruited solely to provide a normative reference for gait parameters and foot-loading patterns. These participants underwent the same TENG–IMU instrumentation and 2 min walk test protocol. The healthy reference data were used for descriptive/illustrative purposes only (e.g., reference plots/bands) and were not included in the formal inferential statistical comparison.

The study was conducted in accordance with the Declaration of Helsinki and received ethical approval from the Institutional Review Board of Nazarbayev University, Astana, Kazakhstan (protocol No. 1009, from 20 December 2024 “Innovative sensor technology application in rehabilitation”). All participants provided written informed consent before enrollment. For participants under 18 years (n = 3), parental consent and participant assent were obtained. Personal data were anonymized (patient_id only), and all procedures minimized risk while ensuring voluntary participation with a right to withdraw at any time.

### 2.2. Categorization of Health Conditions

Self-reported or medical-record histories were manually categorized into seven groups based on anatomical location, injury type, or pathology: spine/back issues (scoliosis, hernia, protrusion, lumbar, spine, vertebrae), knee problems (knee, meniscus, gonarthrosis, ligament), ankle/foot issues (ankle, sprain, flatfeet, plantar fasciitis), hip problems (hip, displacement, dysplasia, labrum), fractures/breaks (fracture, break, fibula, tibia), arthritis/joint issues (arthrosis, arthritis, joint, osteochondrosis) and other. Multimorbid cases were multi-tagged; the prevalence reflects the number of unique patients per category.

Because several participants reported more than one prior musculoskeletal condition, some individuals were represented across multiple descriptive categories. Accordingly, the category prevalence values reflect the number of unique participants assigned to each category rather than mutually exclusive independent groups. Any statistical comparisons across diagnostic categories should therefore be interpreted as exploratory.

### 2.3. TENG Integrated Insole-Shaped Sensor Fabrication

**Figure 1:**
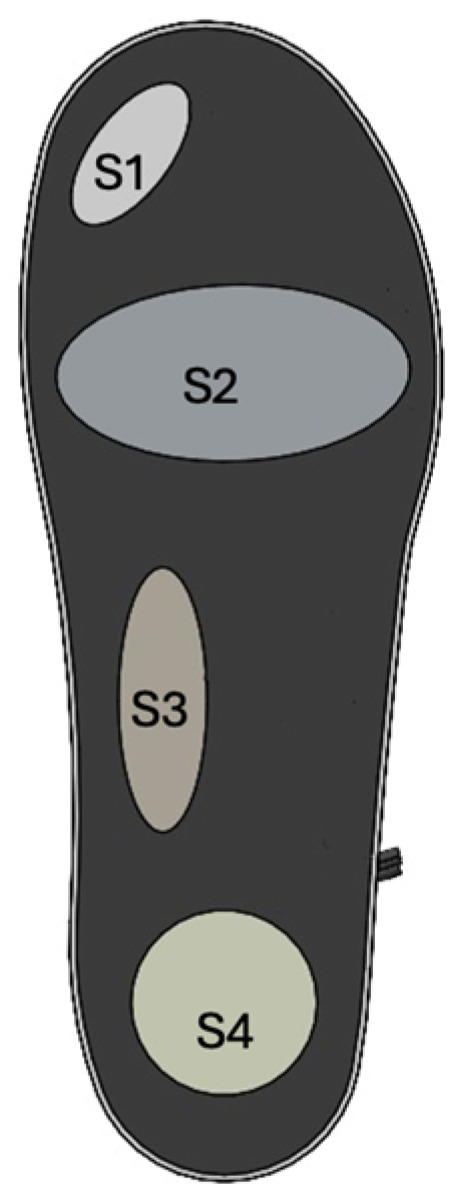
Schematic of the right leg insole showing locations of TENG Sensors 1–4 across plantar regions (big toe—Sensor 1, forefoot—Sensor 2, medial arch—S3, heel—S4).

The TENG sensors were used as insoles, and a detailed preparation methodology has been described elsewhere [16]. A schematic of the smart insole configuration and sensor placement is shown in Figure 1. Four sensing regions were distributed across key plantar contact zones to capture the heel strike, midfoot loading, and forefoot push-off dynamics.

### 2.4. Sensor Testing

For the testing, the four TENG sensors with dimension equal to the electrode region in the insole were prepared, ranging from 9 × 19 mm to 21 × 44 mm and 12 × 30 for the forefoot and midfoot sensors, and a 42 mm diameter circular region for the heel. The TENG sensor performance was tested using equipment like the Tektronix Oscilloscope (TBS1202C, Tektronix, OR, USA) and a customized in-house 3D-printed framed testing system with polyethylene terephthalate (PET) attached to the sensor and a linear motor (PS01-37x120F, LinMot, Spreitenbach, Switzerland) that performed periodic contact with the TENG specimen. A force sensor (Vernier DFS-BTA, Vernier Science Education, OR, USA) was integrated into the system to measure the force applied by the polyester to the tribonegative layer of the TENG. The sensor performance was assessed under different operating conditions, such as the response time, sensitivity, force, and frequency linearity. Every test was repeated at least five times for reliability and consistency.

### 2.5. Hardware Setup and Data Acquisition

**Figure 2:**
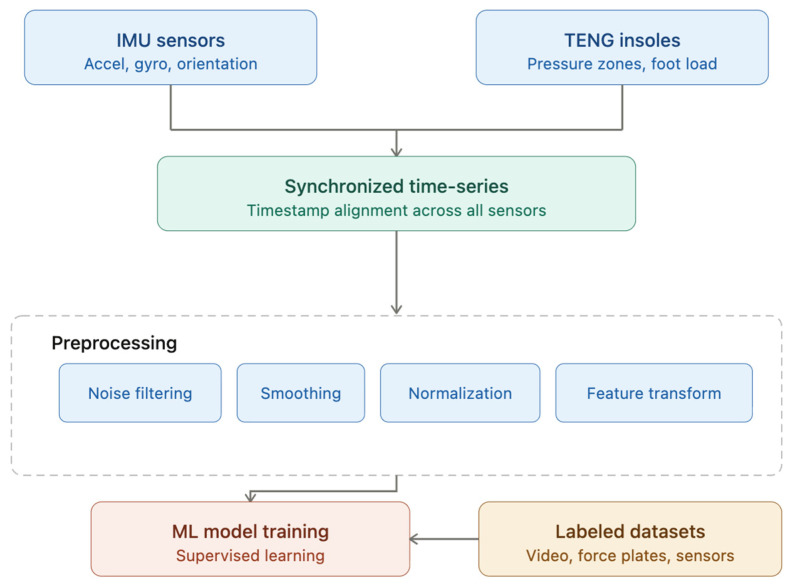
Schematic overview of the multimodal sensor data acquisition and machine learning pipeline.

The sensing system combined two complementary modalities. TENG insoles provided region-specific plantar loading signals reflecting the foot–ground interaction, while IMUs mounted at the ankle and proximal tibia captured lower-limb motion dynamics used to derive temporal gait parameters such as cadence and asymmetry. Data from the IMU sensors and TENG-based insoles were integrated into a unified time series, with all measurements aggregated by a timestamp to ensure temporal alignment across sources. Communication was handled via a Python-based interface that continuously read, parsed, and formatted incoming data streams from the ESP. Prior to model training, a preprocessing pipeline was applied, which may have included noise filtering (median, low-pass, or Savitzky–Golay), signal smoothing, normalization, and feature engineering such as coordinate frame transformations or extraction of derived kinematic descriptors. A custom Python acquisition script managed the data flow, real-time visualization, and recording; the animate function dynamically updated plots with Matplotlib (3.10) and simultaneously saved the decoded data to CSV files suitable for further analysis. The preprocessed data were subsequently used to train supervised machine learning models on annotated datasets, where ground-truth labels were obtained from external reference systems such as video-based motion analysis or force plates. After assembling the hardware system, preliminary walking and stepping tests were conducted. Figure 2 represents the schematic overview on data acquisition, preprocessing and using for ML training. 

### 2.6. Gait Protocol

The active range of motion, ROM, was measured bilaterally immediately pre- and post-2MWT by a trained assessor. Hip flexion/extension, knee flexion, and ankle dorsiflexion/plantarflexion were assessed in standing positions as per standard protocols. The participants performed 3 repetitions per joint; the peak values were recorded to the nearest degree. The participants were asked to wear shoes with insoles of an appropriate size; the IMU sensors were located bilaterally at the ankle (medial malleolus) and at the proximal tibia (1–2 cm inferior to the tibial tuberosity crease). The participants performed a standardized 2 min walk test (2MWT) in a flat, straight 15 m corridor, with indicators at each end, at a self-selected, comfortable speed.

### 2.7. Outcome Measure

Center of mass (COM) displacement: A sensor-derived displacement metric was calculated from the insole signals to summarize the mediolateral (ML) and anteroposterior (AP) distribution of plantar loading during walking. This metric should be interpreted as a COP-like proxy derived from the plantar load distribution rather than a direct estimate of the whole-body center of mass displacement. The metric was obtained by treating each sensor location as a vector on a unit circle and computing the weighted resultant of all sensor vectors at each time point. The sensor weights were defined as the normalized sensor value divided by the sum of all sensor values, yielding the resultant coordinates x and y as the weighted sums of the cosine and sine components of each sensor’s angular position. The resulting x and y time series were used as ML/AP displacement proxies, with larger absolute values indicating greater lateral/anterior shifts in plantar loading.

Data normalization: Raw analog readings for each sensor were normalized by (i) the number of samples and (ii) the maximum analog-to-digital converter value (4095), resulting in values scaled to the [0, 1] range.

Sensor geometry: Eight sensors (four per foot) were positioned according to fixed angular coordinates on a circle (angles specified in Supplementary Information), enabling consistent computation of the resultant loading vector.

*Asymmetry indices*: A global left–right asymmetry index was computed from the summed sensor signals. At each time point, the total plantar signal was defined as the sum of the left and right foot signals. The proportional contribution of each side was calculated (formula in Supplementary Information).

Positive values indicate a relatively greater right-side contribution, whereas negative values indicate a relatively greater left-side contribution. The index was summarized over the walking trial.

*Flatfoot-related loading metrics*: Flatfoot-related features were derived from the distribution of plantar loading across contact regions, quantified from sensor activation and relative load contributions across the forefoot/midfoot/heel regions (and medial–lateral distribution where applicable). These metrics were used to describe patterns consistent with a reduced medial arch support and associated shifts in plantar loading.

### 2.8. Statistical Analysis

The data were analyzed in Python with α = 0.05 (two-tailed). The descriptive statistics included means ± SD, medians, ranges and frequencies. Normality was assessed via a histogram and distribution checks. Associations between gait parameters, age, BMI, and selected clinical features were evaluated using Pearson or Spearman correlations as appropriate. Comparisons across disease-history categories were conducted as exploratory analyses because the subgroup sizes were small, unequal, and partially overlapping due to multimorbidity. Accordingly, the *p*-values from category-based comparisons should be interpreted cautiously and as hypothesis-generating rather than confirmatory.

## 3. Results

### 3.1. Electrical Performance of the Sensor

**Figure 3:**
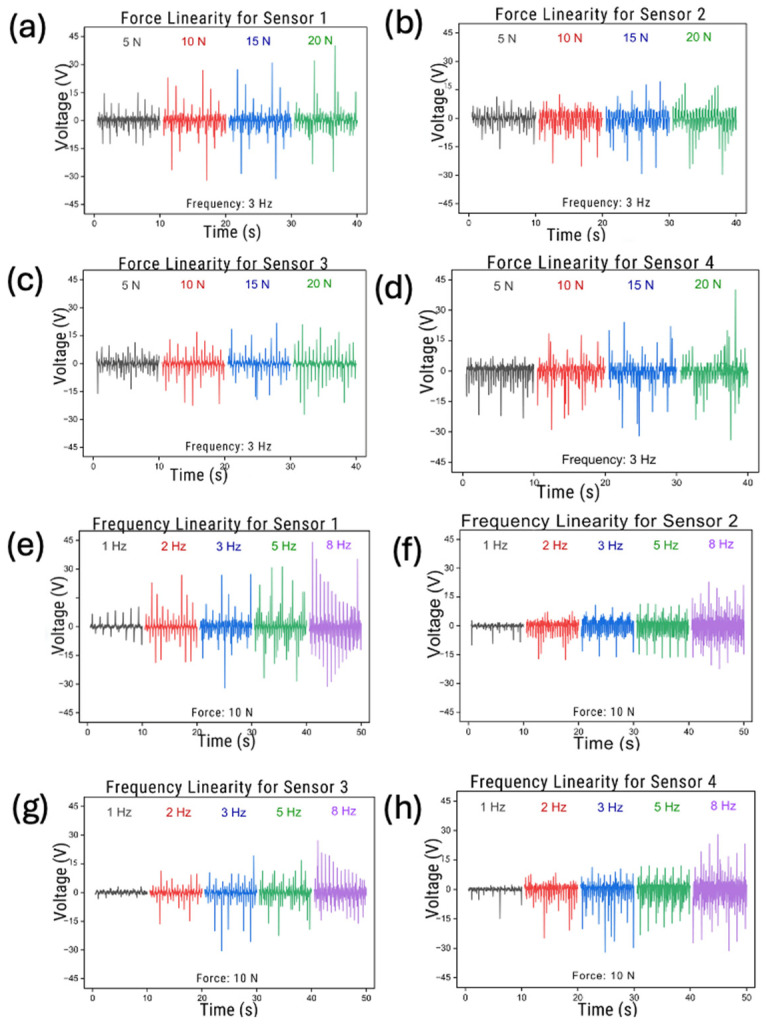
The output performance of the TENG sensor: (**a**–**d**) Force linearity responses of Sensors 1–4 at 5, 10, 15, and 20 N under 3 Hz loading. (**e**–**h**) Frequency linearity responses of Sensors 1–4 at 1, 2, 3, 5, and 8 Hz under 10 N loading.

The force linearity plots for Sensors 1, 2, 3, and 4 at a fixed frequency of 3 Hz showed a clear increase in voltage output with increasing applied force (5, 10, 15, and 20 N), as shown in Figure 3a–d. Larger forces corresponded to a greater contact intimacy and more effective triboelectric charge generation, resulting in higher output amplitudes. All sensors exhibited a monotonic response, confirming force-dependent sensitivity and consistent mechanical–electrical coupling. The repeatability of the peaks across cycles indicates stable electrical behavior during repeated loading.

The frequency linearity plots show the voltage output of all four TENG sensors when driven at 1, 2, 3, 5, and 8 Hz under a constant force of 10 N, as shown in Figure 3e–h. Across all sensors, the voltage amplitude increased with the frequency, indicating that faster contact–separation cycles enhance the charge transfer and output levels. The waveforms also remain periodic and stable, demonstrating that the sensors can reliably track dynamic mechanical input across a wide frequency range. Although the absolute output varies depending on the active surface area of each sensor, the consistent trend across all devices confirms strong frequency-dependent behavior and good responsiveness for real gait conditions, where the footstep frequency typically falls within 1–3 Hz. Among all samples, Sensor 4 (19.6 cm^2^) demonstrated the highest response, producing nearly 10 V at 20 N, whereas Sensor 3 (9.41 cm^2^) generated comparatively lower voltages due to its smaller active region.

Across all sensors, the waveforms showed stable and repeatable signal patterns, with clearly distinguishable responses at each tested condition. These findings confirm that the sensors exhibit a satisfactory linearity in both the force and frequency domains, making them suitable for integration into wearable pressure measurement systems.

**Figure 4:**
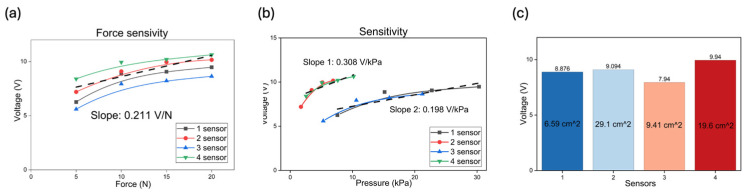
Voltage–pressure characteristics and sensitivity analysis: (**a**) force sensitivity; (**b**) sensitivity curves; and (**c**) voltage versus surface area correlation.

The sensitivity plot shows two distinct linear regimes with slopes of 0.308 V/kPa (low pressure) and 0.198 V/kPa (higher pressure). The higher sensitivity at a low pressure suggests that the sensor is more responsive to small deformations and subtle mechanical stimuli, making it suitable for applications in physiological signal detection and low-force monitoring. The reduction in the slope at higher pressures corresponds to partial saturation as the contact interface approaches full engagement. Larger sensors again perform at higher voltage levels due to their expanded triboelectric area. Figure 4 represents the voltage–pressure behavior and a sensitivity analysis of the TENG sensors. Panel (a) presents the force sensitivity analysis based on the voltage response across the applied loading range, and Panels (b) and (c) illustrate the pressure sensitivity trend and the association between the output voltage and the sensor surface area, respectively.

The bar chart compares the peak voltage output against the effective area of each sensor. A clear positive correlation was observed: sensors with larger surface areas (e.g., Sensor 4 at 19.6 cm^2^) generated higher voltages than smaller sensors (Sensor 1 at 6.59 cm^2^). These results confirm that charge generation in TENGs strongly depends on the available contact area, justifying the use of different sizes in different foot regions. The open-circuit voltage generated by each sensor under identical mechanical loading conditions is shown in Figure 3. As expected, the output voltage exhibited a positive correlation with an increase in the contact area, reaching the highest value of 9.94 V for the largest surface area (19.6 cm^2^). This behavior can be attributed to the greater number of triboelectric charge-generation sites available for contact electrification over larger surface areas. Consequently, a larger charge accumulation during the contact–separation process leads to higher induced potential differences.

### 3.2. Participants and Data Quality

Fifty-three participants (aged 29.3 ± 26.1 years; 22 females, 31 males) were included, with complete gait data from TENG insole and IMU sensors across all sessions. Given the heterogeneous cohort structure, unequal subgroup sizes, and overlapping diagnostic histories, subgroup analyses were treated as exploratory rather than confirmatory. This sample size is comparable to recent wearable sensor studies on gait asymmetry and age effects (N = 20–83).

The BMI distribution showed a higher prevalence of underweight (BMI < 18.5; 36% females vs. 6% males) and extreme obesity (BMI ≥ 35; 23% females vs. 35% males) among females, whereas males were more evenly distributed across the obese categories (Table 2). All sessions yielded sufficient strides for the analysis (mean > 50 valid strides per participant), with no data excluded due to artifacts.

### 3.3. Disease Categorization

Disease histories were categorized anatomically and etiologically based on anatomical location (spine/back, knee, ankle/foot, hip), injury mechanism (fractures/breaks, muscle/tendon), or degenerative processes (arthritis/joint). The trauma and condition histories from 53 participants were categorized into seven groups:

- Spine/Back Issues: scoliosis, hernia, protrusion, lumbar, spine, vertebrae, etc.
- Knee Problems: knee, meniscus, gonarthrosis, ligament, etc.
- Ankle/Foot Issues: ankle, sprain, flatfeet, plantar fasciitis, etc.
- Hip Problems: hip, displacement, dysplasia, labrum, etc.
- Fractures/Breaks: fracture, break, fibula, tibia, etc.
- Arthritis/Joint Issues: arthrosis, arthritis, joint, osteochondrosis, etc.
- Other: anything that does not match the above categories.

Spine/back issues (22 patients, 41.5%) and ankle/foot issues (19 patients, 35.8%) were most prevalent, followed by knee problems (16 patients, 30%). Fractures and breaks affected seven patients (13.2%). Hip problems affected six patients (11.3%), while arthritis/joint issues and the other category each included three patients.

**Figure 5:**
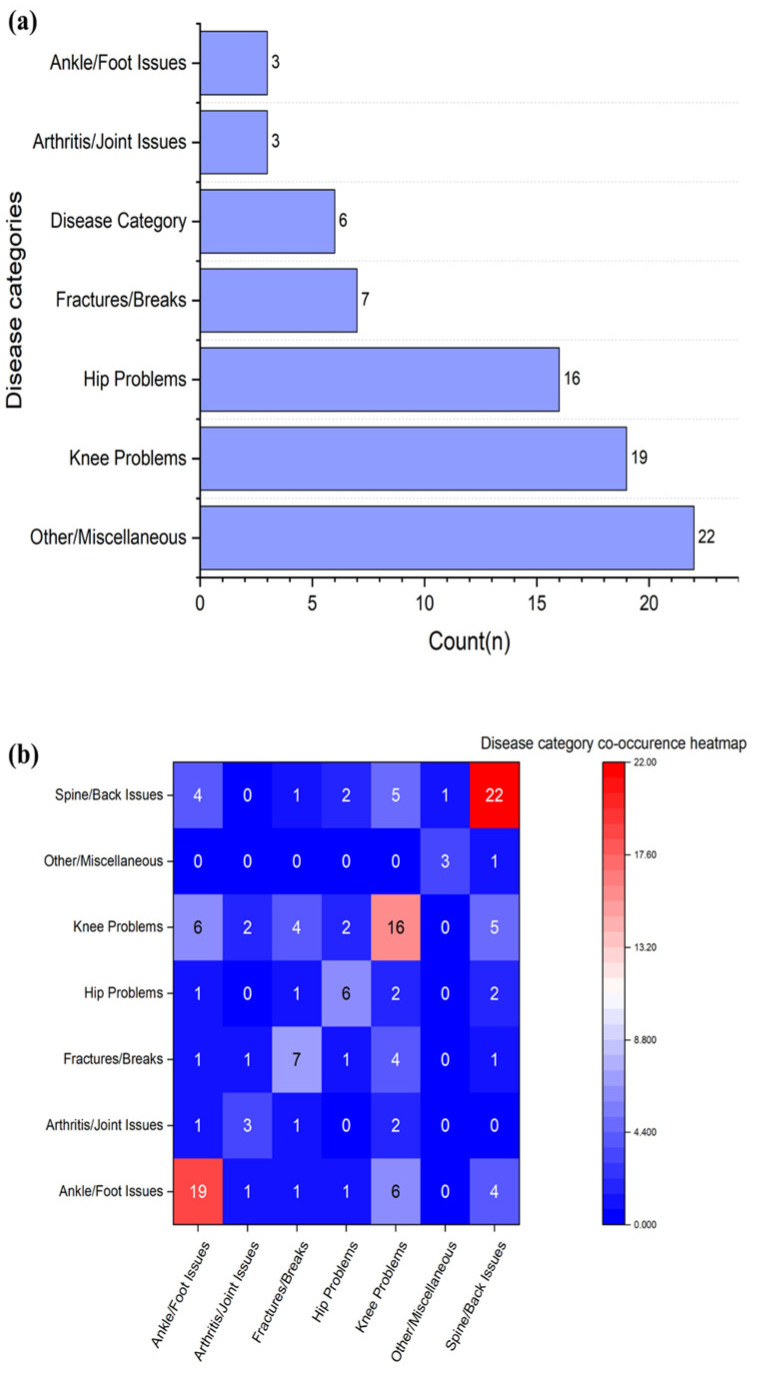
(**a**) Number of patients by disease category. (**b**) Disease category co-occurrence heatmap.

Because patients had several musculoskeletal conditions in their past medical history, musculoskeletal multimorbidity was also analyzed. The average number of categories per patient was 1.49. Figure 5a shows patients grouped by the number of disease categories. Most patients (62.3%) belonged to a single disease category, while 37.7% had multimorbidity (2+ categories). One patient belonged to four categories. Figure 5b shows the heatmap of disease categories.

### 3.4. Temporospatial Parameters

**Figure 6:**
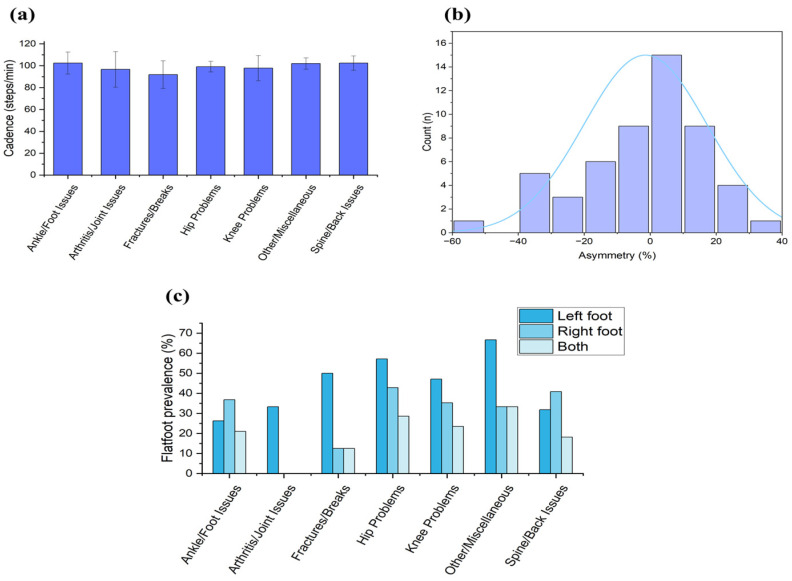
(**a**) Average cadence by disease category, (**b**) asymmetry distribution histogram, and (**c**) flatfoot prevalence by disease category.

The cadence averaged 100.6 ± 10.1 steps/min. Fractures/breaks showed a significant negative association (ρ = −0.23, *p* = 0.043), while the other categories had negligible effects (Table S2 in the Supplementary Information). Figure 6a shows the average cadence by disease category. The cadence declined significantly with age (r = −0.306, *p* = 0.026), dropping from 103.7 steps/min (20–27 years) to 95.7 steps/min (≥34 years).

Asymmetry ranged from −54.4% to 31.2% (mean: −1.4 ± 18.7%). No significant differences across categories emerged (ANOVA F = 0.85, *p* = 0.519; Kruskal–Wallis H = 4.61, *p* = 0.466). The Spearman correlations were weak and non-significant (max ρ = 0.15 for hip/ankle–foot issues), as shown in Table S1 in the Supplementary Information. The asymmetry values followed a normal distribution pattern; Figure 6b presents the asymmetry distribution across participants.

The flatfoot prevalence was 54.7% overall (58.06% males, 50.0% females), as shown in Table S3 in the Supplementary Information. The longitudinal arch indices were low (left: 14.1 ± 11.5%, right: 12.9 ± 10.1%), with lateral dominance (44–46%) (Table S4). Figure 6c summarizes the flatfoot prevalence across categories. Flatfoot features were associated with lateral shifts in the loading proxy during gait (Tables S3 and S4).

### 3.5. Gait-Pattern Differences Across Categories

**Figure 7:**
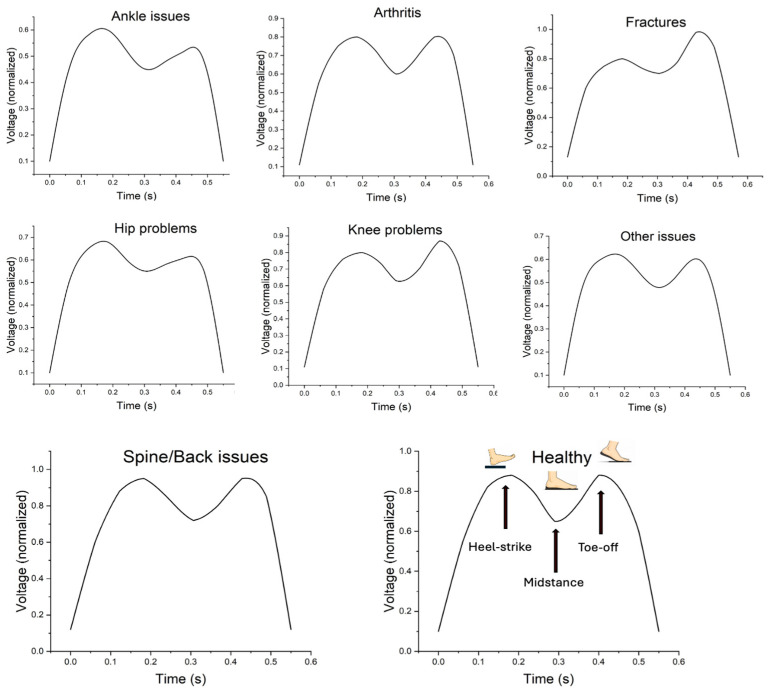
Representative normalized waveform patterns across disease-history categories. The plotted signals represent composite normalized outputs derived from Sensors 1–4 and are intended to visualize the overall gait-pattern morphology within each subgroup. The figure should not be interpreted as a direct comparison of the absolute amplitudes of individual sensors. Sensor-specific amplitude differences were analyzed separately and are described in the main text.

Exploratory differences in the waveform amplitude distributions were observed across history categories for all four sensors (Kruskal–Wallis, *p* < 0.001). However, these findings should be interpreted cautiously given the small and overlapping subgroup structure. Post hoc pairwise comparisons were performed for each sensor; however, given the number of contrasts, these results are summarized in the Supplementary Information. In descriptive terms, Sensor 2 yielded the highest mean amplitudes across most categories, Sensor 3 yielded the lowest, and Sensor 4 showed the greatest variability, particularly in the hip-history subgroup. These findings describe the individual sensor-level amplitude behavior and should be interpreted cautiously given the small and overlapping subgroup structure. Figure 7 presents representative normalized waveform patterns generated by combining the outputs from Sensors 1–4. It is intended to compare the overall gait-pattern morphology across disease-history categories rather than to provide a direct comparison of individual sensor amplitudes.

## 4. Discussion

Recent studies using these sensors describe excellent opportunities for out-of-lab and real-world evaluations of gait parameters, as the healthcare system favors use of durable, safe, biocompatible, relatively low-cost sensor technologies [17]. TENG flexibility allows integration into thin insoles without altering natural gait mechanics, while energy harvesting from foot strikes eliminates the need for batteries, which is critical for prolonged monitoring in rehabilitation and fall-risk assessments. So, the production of a high output and a fast response makes it an emerging technology that bridges the gap between laboratory precision and real-world clinical utility. Current commercial alternatives based on piezoelectric sensors face limitations, including a high cost, a limited durability, signal drift, and excessive power consumption, that hinder clinical adoption [17]. For TENG, its self-charging capability is particularly relevant in wearable settings, where routine activities such as walking or breathing can provide sufficient mechanical input to power sensing and, when coupled with energy storage elements (e.g., capacitors) and charge management components, sustain system operation in a way that aligns with green-energy principles [7]. The combination of plantar sensing (TENG) and motion sensing (IMU) provides complementary biomechanical information that would not be obtainable from either modality alone.

The TENG insoles demonstrated stable, reproducible waveform acquisition under gait-relevant loading conditions, with the output increasing with an applied load and step-frequency behavior consistent with contact-separation triboelectric sensing. Importantly, clear signals were obtained across typical walking frequencies (≈1–3 Hz), supporting the feasibility of battery-free, out-of-lab gait monitoring under intermittent, variable loading. In this cross-sectional rehabilitation cohort, combining TENG-derived plantar loading signatures with ankle/knee IMUs enabled rapid gait phenotyping during a brief 2 min walk test, capturing interpretable temporal parameters and individual-specific asymmetry patterns alongside a high prevalence of arch-related loading features. Together, these findings suggest that self-powered insole sensing can complement IMU-based spatiotemporal assessments and provide additional information on the foot-loading distribution in clinically heterogeneous populations.

### 4.1. TENG-IMU Feasibility

The cohort captured a broad range of body sizes and trauma-related histories, reflecting the heterogeneity typically encountered in rehabilitation settings. Despite this variability, the combined TENG–IMU setup enabled rapid acquisition of gait-relevant signals during a brief 2 min walk test, supporting feasibility for out-of-lab gait phenotyping. The inclusion of a small healthy reference dataset further aided qualitative interpretation of the waveform morphology and loading signatures, without implying between-group inference. The temporal asymmetry showed substantial inter-individual variability across the cohort, but did not differ statistically across history categories (*p* > 0.05). Given the small and overlapping subgroups, these analyses are likely underpowered for group-level inference. Nonetheless, the wide range of asymmetry suggests that the TENG-IMU approach is sensitive to side-dominant gait patterns at the individual level, supporting its use for patient-specific phenotyping and hypothesis generation for future prospectively designed studies.

### 4.2. Age Association

The cadence declined with age (r = −0.306, *p* = 0.026), with an ~8 steps/min difference between the younger (20–27 years) and older (≥34 years) quartiles. This direction is consistent with prior work reporting age-related reductions in walking cadence and temporal performance [18], supporting construct validity of the measurements. The observed age effect is particularly relevant for brief clinical protocols such as the 2MWT, where cadence can serve as a pragmatic marker of the gait capacity in heterogeneous rehabilitation populations.

Participants with a fracture/break history exhibited a lower cadence than participants belonging to other categories, which may reflect residual adaptations following a prior injury. However, given the subgroup size, overlapping histories, and cross-sectional design, these findings should be interpreted cautiously. Future longitudinal work is needed to determine whether sensor-derived cadence changes track recovery trajectories and clinically meaningful outcomes in post-traumatic rehabilitation.

Flatfoot-related loading features were frequent in this cohort (≈55%), which may reflect the clinical setting and the relatively high mean BMI. While prevalence estimates vary by definition and population, the observed frequency is consistent with reports linking flatfoot features to a higher body mass and musculoskeletal burden [19]. In this study, flatfoot features were primarily used to contextualize plantar loading patterns rather than to infer a diagnosis.

### 4.3. Exploratory Loading

The sensor-derived plantar loading distribution metrics (interpreted as a CoP-like proxy) suggested lateral shifts in participants with bilateral flatfoot features, alongside reduced longitudinal arch indices. These findings should be viewed as exploratory and hypothesis-generating, as the metric reflects the plantar load distribution rather than the whole-body center of mass, and the flatfoot classification was based on sensor thresholds without imaging confirmation. Nevertheless, the ability to capture region-specific loading signatures supports the potential utility of TENG insoles for foot-type phenotyping within gait assessments.

### 4.4. Condition-Specific Patterns

Condition-specific waveform and plantar-loading signatures were observed across trauma-history categories, although these patterns should be interpreted as exploratory given the small, overlapping subgroups and potential confounding by multimorbidity. In participants with a spine/back history, higher early-cycle peaks and altered loading transitions were observed. One possible biomechanical explanation is altered proximal control or a weight-transfer strategy associated with spinal conditions. However, trunk and pelvic kinematics were not directly measured in this study. Therefore, these interpretations should be considered hypothesis-generating rather than direct findings. Previous work has shown that spinal pathology and pain-related guarding can reduce sagittal-plane lumbar motion and axial rotation, with compensatory adjustments in frontal-plane control and distal kinematics to maintain stability and limit lumbar loading [20]. While our study did not directly quantify trunk kinematics or lumbar moments, the observed insole-derived signatures are consistent with the notion that altered proximal control can translate into region-specific differences in plantar loading during early stance.

In ankle/foot history, the tendency toward greater lateral heel loading during early stance (Figure 7) aligns with reports that ankle instability is associated with lateralized loading strategies during weight acceptance, including lateral center-of-pressure deviation and altered shock-absorption positioning [21]. Such lateral heel dominance may represent a compensatory response to perceived instability, and the TENG insole’s ability to capture this early-stance signature supports its sensitivity to clinically meaningful foot-loading phenotypes.

For knee-history participants, we observed relatively greater forefoot loading compared with heel strike (Figure 7). This pattern is compatible with the plantar pressure redistribution observed in knee osteoarthritis cohorts, in which an inter-limb loading imbalance and foot posture asymmetry accompany a slower gait and altered temporal characteristics [22]. Although our cohort was defined by a history of trauma rather than standardized KOA grading, the consistency in loading redistribution suggests that TENG-derived signatures may help characterize compensatory gait strategies in individuals with a knee-related musculoskeletal burden.

Participants with a fracture/break history displayed a lower cadence in the main analyses and showed a tendency toward increased relative forefoot loading compared with the heel (Figure 7). Post-fracture gait adaptations are frequently reported, even beyond the acute pain phase [23]; however, in our cohort, no overt limping was observed during testing, and acute pain was an exclusion criterion. Therefore, the loading shifts seen here should be interpreted as potential residual adaptations in the load transfer and push-off strategy rather than as evidence of a limping gait per se.

In the arthritis/joint history subgroup, the foot-loading waveform appeared closer to a symmetric biphasic pattern, similar to the healthy reference signature. This may reflect mixed joint involvement (e.g., hip+knee or knee+ankle) and the very small subgroup size, limiting inference. Finally, hip-history participants showed patterns that may be compatible with prior reports of altered midfoot and heel loading in hip osteoarthritis and compensatory strategies affecting distal pressure distribution [24,25].

Overall, these condition-linked signatures support the potential of self-powered TENG insoles to capture region-specific plantar-loading phenotypes, but they require confirmation in larger, prospectively designed cohorts with standardized clinical characterization.

### 4.5. Limitations

Several limitations should be acknowledged. First, the benchtop sensor characterization experiments were designed to assess the relative electrical behavior (e.g., repeatability, sensitivity, force/frequency response) under controlled loading conditions rather than to fully reproduce the physiological plantar forces encountered during gait. Future studies should evaluate the sensor performance under higher dynamic loads representative of real walking and running conditions.

In addition, the cross-sectional design precludes causal inference. The subgroup analyses were based on small, unequal, and partially overlapping diagnostic categories, limiting the statistical power and independence of observations. The trauma history was partly based on self-reporting and lacked standardized injury grading. Flatfoot features were derived from sensor thresholds without imaging confirmation. Finally, the standardized 2MWT may not reflect free-living gait variability. Despite these limitations, the study supports the feasibility of a TENG-IMU assessment for pragmatic gait phenotyping and motivates larger prospective validation against clinical outcomes.

## 5. Conclusions

This study provides preliminary feasibility evidence that self-powered triboelectric (TENG) insole sensors combined with lower-limb IMUs can capture gait-related temporal metrics and plantar-loading patterns during a brief 2 min walk test in a heterogeneous rehabilitation cohort. The observed associations between the cadence and age, together with individual-specific asymmetry profiles, suggest potential utility for pragmatic gait phenotyping in clinical settings. Importantly, the derived displacement metrics should be interpreted as proxies of the plantar-loading distribution rather than direct estimates of the whole-body center of mass motion. Because the subgroup findings were exploratory, larger prospective studies with standardized clinical characterization are required to validate these sensor-derived phenotypes and determine their role in rehabilitation monitoring and individualized intervention planning.

## Figures and Tables

**Table 1:** Demographic characteristics of participants.

	Female (n = 22)	Male (n = 31)
Height (m)	1.63 ± 0.08	1.79 ± 0.07
Body mass (kg)	66.3 ± 18.3	75.9 ± 11.9
Age	34 ± 17.5	27 ± 8.7

**Table 2:** BMI categorization of patients (n = 53).

	Female	Male
Underweight (BMI < 18.5)	8	2
Normal (18.5–24.9)	6	5
Overweight (25.0–29.9)	1	3
Obese (30.0–34.9)	2	10
Extremely obese (≥35.0)	5	11

## Data Availability

The data are available upon request.

## Supplementary Materials

The following supporting information can be downloaded at: https://www.mdpi.com/article/10.3390/s26103191/s1, Table S1. Analysis of asymmetry across different disease categories, Table S2. Analysis of cadence across different disease categories, Table S3: Prevalence of flatfoot by Gender, Table S4: Mean prevalence of flatfoot.

## Abbreviations

The following abbreviations are used in this manuscript:

TENG	triboelectric nanogenerator
IMU	inertial measurement unit
ESP	Espressif Systems
KOA	knee osteoarthritis
COM	center of mass
ROM	range of motion

## References

[1] Hulleck A.A., Menoth Mohan D., Abdallah N., El Rich M., Khalaf K. (2022). Present and future of gait assessment in clinical practice: Towards the application of novel trends and technologies. Front. Med. Technol..

[2] Auvinet B., Berrut G., Touzard C., Moutel L., Collet N., Chaleil D., Barrey E. (2003). Gait abnormalities in elderly fallers. J. Aging Phys. Act..

[3] Kumar R., Bogia P., Singh V., Reddy T.O. (2025). The running gait analysis technology: A comprehensive systematic literature review. J. Orthop..

[4] Tao W., Liu T., Zheng R., Feng H. (2012). Gait analysis using wearable sensors. Sensors.

[5] Simon S.R. (2004). Quantification of human motion: Gait analysis—Benefits and limitations to its application to clinical problems. J. Biomech..

[6] Sethi D., Bharti S., Prakash C. (2022). A comprehensive survey on gait analysis: History, parameters, approaches, pose estimation, and future work. Artif. Intell. Med..

[7] Meng S., McErlain-Naylor S.A., Dharmasena R.D.I.G. (2025). Wearable triboelectric nanogenerators for biomechanical sensing. Nano Energy.

[8] Burnie L., Chockalingam N., Holder A., Claypole T., Kilduff L., Bezodis N. (2023). Commercially available pressure sensors for sport and health applications: A comparative review. Foot.

[9] Ali S.M., Noghanian S., Khan Z.U., Alzahrani S., Alharbi S., Alhartomi M., Alsulami R. (2025). Wearable and flexible sensor devices: Recent advances in designs, fabrication methods, and applications. Sensors.

[10] Zhu Q., Wu T., Wang N. (2023). From piezoelectric nanogenerator to non-invasive medical sensor: A review. Biosensors.

[11] Zhang Q., Jin T., Cai J., Xu L., He T., Wang T., Tian Y., Li L., Peng Y., Lee C. (2022). Wearable triboelectric sensors enabled gait analysis and waist motion capture for IoT-based smart healthcare applications. Adv. Sci..

[12] Liu L., Shi Q., Lee C. (2021). A hybridized electromagnetic-triboelectric nanogenerator designed for scavenging biomechanical energy in human balance control. Nano Res..

[13] Liu L., Li J., Tian Z., Hu X., Wu H., Chen X., Zhang L., Ou-Yang W. (2024). Self-powered porous polymer sensors with high sensitivity for machine learning-assisted motion and rehabilitation monitoring. Nano Energy.

[14] Demircan E., Yi C., Harris E., Khoo I.-H. (2025). Comparative evaluation of triboelectric nanogenerator-based and inertial measurement unit-based systems for smart gait analysis in human activity recognition. J. Micro-Bio Robot..

[15] Parashar P., Sharma M.K., Nahak B.K., Khan A., Hsu W.-Z., Tseng Y.-H., Chowdhury J.R., Huang Y.-H., Liao J.-C., Kao F.-C. (2025). Machine learning-driven gait-assisted self-powered wearable sensing: A triboelectric nanogenerator-based advanced healthcare monitoring. J. Mater. Chem. A.

[16] Issabek M., Oralkhan S., Anash A., Nurbergenova N., Balapan A., Yeshmukhametov A., Rakhmanov Y., Kalimuldina G. (2024). AI-enhanced gait analysis insole with self-powered triboelectric sensors for flatfoot condition detection. Adv. Mater. Technol..

[17] Zhao C., Jin Y., An R., Uchitomi H., Miyake Y. (2025). Deep learning-based extension of gait segmentation to abnormal patterns using inertial measurement units. Eng. Appl. Artif. Intell..

[18] Aguiar E.J., Schuna J.M., Barreira T.V., Mire E.F., Broyles S.T., Katzmarzyk P.T., Johnson W.D., Tudor-Locke C. (2019). Normative peak 30-min cadence (steps per minute) values for older adults: NHANES 2005–2006. J. Aging Phys. Act..

[19] Pita-Fernandez S., Gonzalez-Martin C., Alonso-Tajes F., Seoane-Pillado T., Pertega-Diaz S., Perez-Garcia S., Seijo-Bestilleiro R., Balboa-Barreiro V. (2017). Flat foot in a random population and its impact on quality of life and functionality. J. Clin. Diagn. Res..

[20] Zhang Z., Zou J., Lu P., Hu J., Cai Y., Xiao C., Li G., Zeng Q., Zheng M., Huang G. (2024). Analysis of lumbar spine loading during walking in patients with chronic low back pain and healthy controls: An OpenSim-based study. Front. Bioeng. Biotechnol..

[21] Elabd O.M., Elabd A.M., El-Aez M.S.A., Taha M.M., Mohammed A.H. (2024). Impact of chronic ankle instability on gait loading strategy in individuals with chronic ankle instability: A comparative study. J. Neuroeng. Rehabil..

[22] Wang Y., Zhang P., Chen G., Jiang T., Zou Y. (2024). Comparison of the asymmetries in foot posture, gait and plantar pressure between patients with unilateral and bilateral knee osteoarthritis based on a cross-sectional study. Sci. Rep..

[23] Rauer T., Friedl E., Gamble J.G., Zelle B.A., Pape H.-C., Pfeifer R. (2023). Long-term analysis of chronic pain associated with lower extremity injuries. Arch. Orthop. Trauma Surg..

[24] Pawłowska K.M., Pawłowski J., Grochulska A. (2024). The distribution of pressure forces of the foot on the ground during gait in patients with hip osteoarthritis. J. Back Musculoskelet. Rehabil..

[25] Cichy B., Wilk-Frańczuk M. (2006). Gait analysis in osteoarthritis of the hip. Med. Sci. Monit..

