# Supplementary material for: Self-Powered Triboelectric Insole for Gait Asymmetry and Plantar Pressure Signatures in Rehabilitation Patients: A Cross-Sectional Study
Source: Sensors (Basel). 2026 May 18;26(10):3191. doi: 10.3390/s26103191 (PMC13210678; doi:10.3390/s26103191)
Supplement: Supplementary file 1 [file sensors-26-03191-s001.zip › sensors-4272864-supplementary.pdf]

Supplementary Information

Self-powered triboelectric insole sensors for gait asymmetry and plantar pressure signatures in rehabilitation patients: a cross-sectional study

Methods:

Sensor angles (in degrees):

Sensor placement angles for calculating the center of mass (in degrees) # Order: Left S1, S2, S3, S4, Right S1, S2, S3, S4 SENSOR_ANGLES = np.array([247.5, 202.5, 157.5, 112.5, 292.5, 337.5, 22.5, 67.5])

Vector sum:

Each sensor is represented as a vector on a circle.

The weight of each vector = the normalized sensor value / the sum of all values.

The X and Y components are calculated:

$$x = \sum_{i=1}^{8} (\cos \alpha \ \times W)$$

$$y = \sum_{i=1}^{8} (\sin \alpha \times W)$$

Asymmetry indices:

A global left-right asymmetry index was computed from the summed sensor signals. At each time point, the total plantar signal was defined as the sum of left and right foot signals. The proportional contribution of each side was calculated as:

A_L = \frac{L}{L+R}, \quad A_R = \frac{R}{L+R},

and the asymmetry index was defined as:

AI = A_R - A_L.

Table S1. Analysis of asymmetry across different disease categories

Disease Category	Spearman ρ	p-value
Spine/Back Issues	−0.059	0.604

Ankle/Foot Issues	+0.153	0.175
Knee Problems	−0.116	0.304
Fractures/Breaks	+0.009	0.937
Arthritis/Joint Issues	−0.098	0.386
Hip Problems	+0.152	0.177
Other/Miscellaneous	−0.103	0.365

Table S2. Analysis of cadence across different disease categories

Disease Category	Spearman ρ	p-value	Interpretation
**Spine/Back Issues**	+0.168	0.137	weak ↑ trend
**Ankle/Foot Issues**	+0.076	0.504	negligible
**Knee Problems**	−0.064	0.571	negligible
**Fractures/Breaks**	−0.227	0.043	significant ↓
**Arthritis/Joint Issues**	−0.010	0.930	none
**Hip Problems**	−0.066	0.560	negligible
**Other/Miscellaneous**	+0.039	0.734	negligible

Table S3: Prevalence of flatfoot by Gender

Gender	Total	With Flatfoot	Prevalence %
Male	31	18	58.06%
Female	22	11	50.00%
Overall	53	29	54.72%

Table S4: Mean prevalence of flatfoot

Variable	Mean ± SD	Median	Range
BMI (kg/m²)	30.61 ± 13.92	30.09	13.87 - 37.43
Height (cm)	172.41 ± 10.88	173.00	146 - 194
Weight (kg)	71.14 ± 15.78	73.00	45 - 112
Left Foot - Longitudinal Arch (%)	14.07 ± 11.45	11.00	1 - 44
Left Foot - Lateral Arch (%)	44.59 ± 13.03	49.00	1 - 62

Right Foot - Longitudinal Arch (%)	12.90 ± 10.10	10.00	0 - 36
